# Characterization of Rhizosphere Microbial Communities for Disease Incidence and Optimized Concentration of Difenoconazole Fungicide for Controlling of Wheat Dwarf Bunt

**DOI:** 10.3389/fmicb.2022.853176

**Published:** 2022-05-09

**Authors:** Huanyu Jia, Ghulam Muhae-Ud-Din, Han Zhang, Qianqian Zong, Sifeng Zhao, Qingyuan Guo, Wanquan Chen, Li Gao

**Affiliations:** ^1^State Key Laboratory for Biology of Plant Diseases and Insect Pests, Institute of Plant Protection, Chinese Academy of Agricultural Sciences, Beijing, China; ^2^Key Laboratory at Universities of Xinjiang Uygur Autonomous Region for Oasis Agricultural Pest Management and Plant Protection Resource Utilization, Shihezi University, Xinjiang, China; ^3^Department of Agricultural Science, Xinjiang Agricultural University, Ürümqi, China

**Keywords:** rhizosphere microorganisms, wheat dwarf bunt, *Tilletia controversa*, difenoconazole, disease incidence

## Abstract

Rhizosphere soil microorganisms have great agricultural importance. To explore the relationship between rhizosphere microorganisms and the disease incidence, and to optimize the concentration of difenoconazole fungicide for the control of wheat dwarf bunt, caused by *Tilletia controversa* Kühn, the rhizosphere microorganisms were characterized based on sequencing methods. We found that the disease incidence correlated with the relative abundance of some microbial communities, such as Acidobacteria, Nocardioides, Roseiflexaceae, Pyrinomonadaceae, and Gemmatimonadaceae. Actinobacteria showed significant differences in the infected soils when compared to the control soils, and the relative abundance of Acidobacteria, Pyrinomonadaceae, Gemmatimonadaceae, and Saccharimonadales populations was distinctly higher in the *T. controversa*-inoculated group than in the control group. The members of Dehalococcoidia, Nitrosomonadaceae, and Thermomicrobiales were found only in *T. controversa*-inoculated soils, and these taxa may have potential effects against the pathogen and contribute to disease control of wheat dwarf bunt. In addition, for *T. controversa*-infected plants, the soil treated with difenoconazole showed a high relative abundance of Proteobacteria, Actinobacteria, Ascomycota, Basidiomycota, Mortierellomycota, and Olpidiomycota based on the heatmap analysis and ANOVA. Our findings suggest that the optimized concentration of fungicide (5% recommended difenoconazole) exhibits better control efficiency and constant diversity in the rhizosphere soil.

## Introduction

Dwarf bunt of wheat is caused by *Tilletia controversa* Kühn and is an economically devastating disease of wheat (Liu et al., 2020), which not only causes 80% of total yield loss but also affects the milled flour quality with a stinky smell (Lu et al., 2005). The use of seeds coated with fungicide is an effective and convenient method to control the dwarf bunt disease (Shakoor et al., 2014; Din et al., 2021). The increasing demand for the quality of foods worldwide emphasizes the need to develop better friendly strategies for the efficient management of dwarf bunt disease.

Microbial diversity and composition play an important role in multiple soil functions and in improving soil fitness and fertility (Fuhrman, 2009; Maestre et al., 2015). Microbial abundance is used as an indicator for assessing soil quality (Ravindran and Yang, 2015). Almost all parts of the plant interact with microbes during the growth and developmental period and the plants discharge various compounds to feed and attract the associated microbes. The microbes also discharge various substances that favor plant physiological and morphological functions, increase the resistance level against malignant microbes, and increase plant strength, thus allowing plants to tolerate abiotic and biotic stress conditions (Schirawski and Perlin, 2018). The microbes present in the soil not only influence the physiology of plants but also alter different morpho-physiological traits of plant tissues, resulting in a reduction in the yields or quality of the cultivated product (Bezemer and Van Dam, 2005; Wardle et al., 2011). The population of these microorganisms is influenced directly or indirectly by other beneficial mutualistic microbes or pathogens (Rudrappa et al., 2010). Some studies have demonstrated that plant hosts and their growth and developmental stages have a significant impact on the rhizospheric microbiome (Chaparro et al., 2013; Peiffer et al., 2016). The rhizospheric microbes can enhance disease resistance in plants, thus protecting the plant from the development of disease (Kwak et al., 2018; Carrión et al., 2019). For instance, several rhizospheric microbes are known to exhibit antagonistic effects against pathogens, such as *Trichoderma* spp., *Bacillus* spp., *Rhizobia* spp., *Lactobacilli* spp., *Pseudomonas* spp., and *Gliocladium* spp. (Fravel, 2005; Nuzzo et al., 2020). Increased microbial diversity in the rhizospheric region may improve the disease resistance of crops against pathogen attack (Mendes et al., 2011; Zhou et al., 2019). The high *Pseudomonas* diversity increased pathogen destruction through microbial communities and competition with the plant pathogen (Hu et al., 2016). The bacterial community significantly affects the population of *Fusarium verticillioides* pathogen in the maize crop (Niu et al., 2017). *F. oxysporum* f. sp. *lycopersici* changes the composition of microbiomes in tomato crops and alters the relative abundance of the microbial community that acts as biocontrol agents (Zhou et al., 2020). Many plant-associated microbes, such as *Pseudomonas* spp., *Trichoderma* spp., *Bacillus* spp., *Rhizobia* spp., *Lactobacilli* spp., and *Gliocladium* spp., act as potential biocontrol agents against *Fusarium* spp. (Hu et al., 2016; Niu et al., 2017).

Fungicide use can pose a serious threat to the natural environment, in particular soil, by adversely affecting the soil microorganisms and biochemical processes (Banks et al., 2005; Wightwick et al., 2013). Fungicides exert a negative effect on non-target beneficial microorganisms (Guo et al., 2015). Baćmaga et al. (2016) reported that Falcon 460 EC fungicide reduces the population density of bacterial (*Bacillus* spp.) and fungal (*Penicillium* and *Rhizopus* spp.) species. Kalia and Gosal (2011) reported that the use of pesticides reduces the soil microbiome population in rice–wheat cropping system. All these results suggest that a diverse microbial community could affect the establishment, survival, and functioning of plant pathogens and play a significant role in disease suppression (Mendes et al., 2011).

In this study, to explore the effect of rhizosphere microbial communities on the disease incidence and to optimize the concentration of difenoconazole fungicide for controlling wheat dwarf bunt caused by *T. controversa*, 16 wheat varieties were inoculated with *T. controversa*, and a highly susceptible wheat cultivar (Morocco) coated with six different concentrations of difenoconazole was used to optimize the rhizosphere microbial community. To our knowledge, this is the first study to determine the potential of some rhizosphere soil microbial communities to provide protection against *T. controversa* and to optimize the effective dose of difenoconazole based on the rhizosphere microorganisms in *T. controversa*-infected and non-infected wheat plants.

## Materials and Methods

### Plant Materials and Treatments

Sixteen wheat varieties (Table 1A) and one highly susceptible variety (Morocco) to *T. controversa* were collected from the Institute of Plant Protection, Chinese Academy of Agricultural Sciences, China. Wheat kernels were surface sterilized with 30% NaOCl for 5 min, washed five times with ddH_2_O, and grown for 30 days in an incubator at 5°C (AUCMA, Qing Dao, China) to induce the vernalization process. After vernalization, wheat seedlings were sown in a 2:2 ratio of soil and organic matter in pots (diameter 23 cm and height 15 cm). About 10–12 seedlings were transplanted into every pot. Five inoculations of *T. controversa* spores were administered into the root zone of all the above-mentioned wheat varieties, with three biological replicates, and three sets of each variety were used as controls. The seeds of the Morocco cultivar were coated with six different concentrations of difenoconazole fungicide, and the details are presented in Table 2.

**TABLE 1A:** List of the varieties used in this study.

Variety name	Control	Infected*	Variety name	Control	Infected*
New Winter 1	NW1_C	NW1_I	New Winter 19	NW19_C	NW19_I
New Winter 4	NW4_C	NW4_I	New Winter 20	NW20_C	NW20_I
New Winter 7	NW7_C	NW7_I	New Winter 24	NW24_C	NW24_I
New Winter 11	NW11_C	NW11_I	New Winter 33	NW33_C	NW33_I
New Winter 12	NW12_C	NW12_I	New Winter 35	NW35_C	NW35_I
New Winter 13	NW13_C	NW13_I	New Winter 46	NW46_C	NW46_I
New Winter 14	NW14_C	NW14_I	New Winter 51	NW51_C	NW51_I
New Winter 17	NW17_C	NW17_I	Yinong 18	YN18_C	YN18_I

* “Infected” refers to the application of T. controversa in the root zone of wheat cultivars. NWI_C and NW1_I refer to the New Winter 1 control and New Winter 1 inoculated plants, respectively.

**TABLE 1B:** Disease incidence in different varieties treated with *T. controversa*.

Variety name	Disease incidence	Variety name	Disease incidence
New Winter 1	–	New Winter 19	–
New Winter 4	10%	New Winter 20	4%
New Winter 7	–	New Winter 24	–
New Winter 11	–	New Winter 33	8%
New Winter 12	–	New Winter 35	4%
New Winter 13	–	New Winter 46	–
New Winter 14	–	New Winter 51	14%
New Winter 17	–	Yinong 18	6%

**TABLE 2:** Seed dressing with difenoconazole application against *T. controversa* infected and non-infected wheat rhizosphere.

Fungicide (ratio)	Dose	Infected	Control
1.5% dose	2.25 mL/100 kg	IA	NG
3% dose	4.5 mL/100 kg	IB	NH
5% dose	7.5 mL/100 kg	IC	NI
Recommended dose	150 mL/100 kg	ID	NJ
1.5 times dose	225 mL/100 kg	IE	NK
No fungicide	No seed treatment	IF	NL

*Wheat grains were coated with difenoconazole fungicide with the different range of concentration with aim to check the effect of different concentration on rhizosphere soil microorganisms in T. controversa infected and control plants. Infected means T. controversa applied in the root zone of seedlings, while control stands for no T. controversa.*

### Collection and Processing of Soil Samples

Soil samples were collected (three biological replicates and four technical replicates) from both *T. controversa*-inoculated and control pots at a depth of 20 cm during the ripening stage of wheat, packed into 50-ml test tubes (Houdior, China), and labeled with a permanent marker. All the impurities were removed (filtered by using a 40-mesh strainer) from the samples in the laboratory for further processing. The fine soil was then weighed, and 20 g of the soil was added into a sterile centrifuge tube. The soil samples were stored at –80°C for further experiments.

### Molecular Detection of *Tilletia controversa*

Plant leaves were collected after 1 week of inoculation with *T. controversa* in the root zone of seedlings. The DNA was extracted, and the sequence characterized amplified region (SCAR) markers were used to determine whether the infection was successful. The design of primers and PCR experiments were performed according to a previous report (Gao et al., 2014).

### Extraction of Total Soil DNA and PCR Amplification

Soil DNA was extracted from every sample using a DNA kit (Omega Bio-tek, Norcross, GA, United States) according to the manufacturer’s instructions by using 0.5 g of soil suspension. The concentration and quality of the extracted DNA were analyzed by using NanoDrop 2000 machine (Thermo Scientific, United States) and adjusting the absorbance wavelength to 260/280 nm and 260/230 nm, respectively. The DNA quality was analyzed by subjecting a 6-μl aliquot of DNA sample to 1% agarose gel electrophoresis. The extracted and purified DNA was kept at –80°C for further use.

### Bacterial and Fungal Gene Amplification

An aliquot of the high-quality DNA extracted from every sample was further used as a template for DNA amplification. The V3–V4 bacterial hypervariable regions of 16S rRNA genes were amplified using the specific primers 338F 5′- ACTCCTACGGGAGGCAGCAG-3′ and 806R 5′- GGACTACHVGGGTWTCTAAT-3′, and PCR reactions were performed according to the method proposed by Tian et al. (2018). Primers ITS3_KYO2 (5′-GATGAAGAACGYAGYRAA-3′) and ITS4 (5′-TCCTCCGCTTATTGATATGC-3′) were used to amplify fungal ribosomal DNA ITS2 gene, and PCR reactions were performed according to Din et al. (2021).

### Illumina MiSeq Sequencing (16S and ITS)

Illumina MiSeq sequencing was performed for the PCR products of bacteria (16S) and fungus (ITS) by following the method of our previous study (Din et al., 2021). The procedure for the construction of the library included the following steps: (1) connecting the “Y”-shaped joints, (2) removing self-ligated fragments using magnetic beads, (3) enriching the library template by PCR amplification, and (4) performing sodium hydroxide denaturation for single-stranded DNA fragments. Sequencing was conducted using the Illumina MiSeq PE300 platform at Meiji Biomedical Tech. Co., Ltd. (Shanghai, China).

### Processing of Sequenced Data

The original sequences were processed for quality control using Trimmomatic software and ligated using FLASH software (Magoč and Salzberg, 2011). Sequences with ambiguous bases were removed. Operational taxonomic units (OTUs) were clustered by using UPARSE software (version 7.1^1^) with 97% similarity as the cut-off. The taxonomic classification was performed using the Ribosomal Database Project (RDP) classifier^2^ (Tian et al., 2018) and compared to the Silva database (SSU123) with a homology of 70% (Xu et al., 2018).

### Statistical Analysis

The α-diversity metric was calculated using Mothur (version v.1.30.1, collect.single command), while β-diversity and other parameters were calculated using QIIME (Caporaso et al., 2011) and R software. Principal component analysis (PCA) was performed to examine the differences between individuals and communities. Further analysis was done to check significant alterations in the community structures in every sample at every classification level. Statistical analyses were done by using R.V.3.2.1 with the VEGAN package. The incidence of dwarf bunt was scored for each variety as follows: Disease incidence = Number of infected heads/total number of heads × 100.

## Results

### Effects of *Tilletia controversa* on Rhizosphere Soil Microbial Community

**FIGURE 1:**
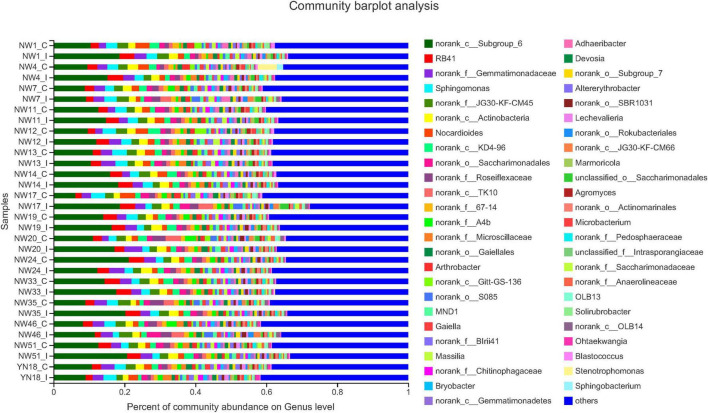
Relative abundance of the dominant rhizosphere soil bacterial in 16 wheat cultivars. The relative abundances are based on the proportional frequencies of the DNA sequences that could be classified. The length of the colored bars indicates the average relative abundance in each sample group. C indicates control cultivar and I indicates infected wheat cultivar.

A total of 3,928,163 high-quality sequences were obtained from all the soil samples, with 34,750–74,834 sequences identified for each sample (average of 61,920 sequences) (Supplementary Table 1). All the infected samples were confirmed by the presence of a specific SCAR band characteristic of the pathogen (Supplementary Figure 1). We found that the dominant phyla were norank_c_Subgroup_6, RB41, norank_f_Gemmatimonadaceae, Sphingomonas, norunk-f_JG30-KF-CM45, norunk_c_Actinobacteria, and Nocardiodes (Figure 1). For specific norunk_c_Subgroup_6, the abundance of all the species was found to be increased after infection, except for New Winter 13 and 24, and Yinong 18 cultivars. For RB41, the abundance of most of the bacteria was increased, except for New Winter 7, 24, 33, and 46 and Yinong 18 cultivars, while the abundance did not show a significant difference between the infected and control plants for New Winter 14, 19, 20, and 51 wheat varieties. For norank_f_Gemmatimonadaceae, the abundance of most of the species increased after the pathogen infection, except for New Winter 7 and 35 and Yinong 18, while no big difference between infected samples and control samples was observed for New Winter 12 and 46 varieties. For Sphingomonas, the abundance of species did not show a significant difference between the wheat varieties when compared to the previous three dominant phyla.

### Effects of Microbial Diversity on Disease Incidence

**FIGURE 2:**
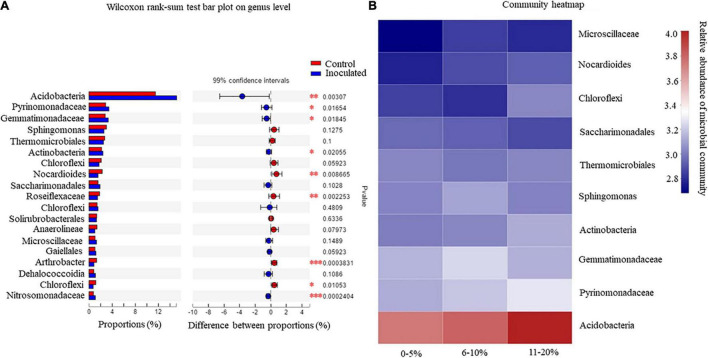
**(A)** Wilcoxon rank-sum test analysis for the rhizosphere soil microorganisms. *P = significant, **P = highly significant, ***P = extremely significant. **(B)** Community heatmap analysis of rhizosphere soil microorganisms.

A Wilcoxon rank-sum test was performed to demonstrate whether *T. controversa* inoculation and control variables influenced the structure of the microbial community (Figure 2A). The levels of significance (*P ≤ 0.05; **P ≤ 0.01; ***P ≤ 0.001) determined by the Wilcoxon rank-sum test were used for comparing the root soil microbial community in *T. controversa*-inoculated and control samples. The results revealed that the populations of *Arthrobacter* sp. and Nitrosomonadaceae were highly significant (*P* ≤ 0.001); Acidobacteria, Nocardioides, and Roseiflexaceae were significantly different (*P* ≤ 0.01); and Pyrinomonadaceae, Gemmatimonadaceae, Actinobacteria, and Chloroflexi were also significantly different (*P* ≤ 0.05).

The rhizosphere microbial community plays an important role in the disease incidence of *T. controversa* in different wheat cultivars. The disease incidence was classified into three levels, that is, 0–5%, 6–10%, and 11–20% (Figure 2B). The disease incidence was correlated with the abundance of some microbial communities based on the heatmap analysis of the community. Acidobacteria showed a direct proportional relationship with disease incidence; as the abundance of Acidobacteria increased from 3.6 to 4.0%, the rate of disease incidence also increased. The same pattern was observed for the members of Pyrinomonadaceae, Actinobacteria, and Nocardioides, that is, as the abundance level of the microbial community increased, the level of disease incidence also increased. Interestingly, in the case of Sphingomonas, the disease incidence increased as the abundance level decreased. Furthermore, the disease incidence was inversely proportional to the abundance of Saccharimonadales. The study results indicated that the disease incidence varied between the cultivars. The maximum disease incidence was recorded in New Winter 51, and the minimum was recorded in New Winter 20 and New Winter 35, with values of 14 and 4%, respectively (Table 1B).

### Microbial Taxonomic Distribution in Different Concentrations of Difenoconazole in Rhizosphere Soil

Seeds coated with six different concentrations of difenoconazole for infected and control samples (three replicates) were used for Illumina MiSeq sequencing using bacteria- and fungi-specific primers. The number of OTUs was high in the fungal (2,028,447) kingdom when compared to the bacterial (605,412) kingdom. The populations of fungal and bacterial phyla were observed in both *T. controversa*-infected and non-infected plant samples obtained from seeds coated with different concentrations of difenoconazole fungicide (Supplementary Tables 2, 3). Furthermore, data were analyzed through the Shannon curve, which reflects the microbial diversity. When the curve tends to be flat, it indicates that the samples contain a sufficient representation of bacterial (Supplementary Figure 2A) and fungal (Supplementary Figure 2B) communities.

**FIGURE 3:**
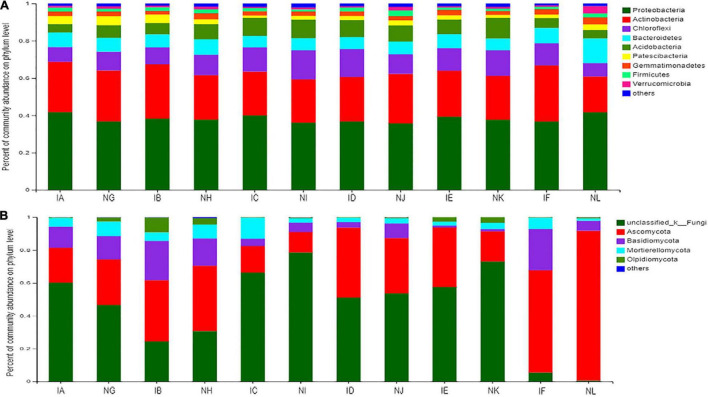
**(A)** Comparison of taxonomic distributions of bacterial phyla between different concentrations of difenoconazole fungicide in *T. controversa*-infected and non-infected samples. IA (*T. controversa* + 1.5% dose of difenoconazole), NG (control + 1.5% dose of difenoconazole), IB (*T. controversa* + 3% dose of difenoconazole), NH (control + 3% dose of difenoconazole), IC (*T. controversa* + 5% dose of difenoconazole), NI (control + 5% dose of difenoconazole), ID (*T. controversa* + recommended dose of difenoconazole), NJ (control + recommended dose of difenoconazole), IE (*T. controversa* + 1. 5 times dose of difenoconazole), NK (control + 1. 5 times dose of difenoconazole), IF (*T. controversa* + no difenoconazole), and NL (control + no difenoconazole). The control indicates treatment with ddH_2_O. **(B)** Comparison of taxonomic distributions of fungal phyla between different concentrations of difenoconazole fungicide in *T. controversa*-infected and non-infected samples.

In Figure 3A, we show nine bacterial phyla from the dataset whose relative abundance reached up to 2% in at least one sample, and the taxonomic distributions are presented with average relative abundances. Among the bacterial phyla, the most abundant phylum was Proteobacteria, with the percentage of community abundance of 0. 42 (42%) in *T. controversa* + 1.5% dose of difenoconazole (IA), *T. controversa* + 5% dose of difenoconazole (IC), *T. controversa* + 1. 5 times dose of difenoconazole (IE), and control + no difenoconazole (NL). The percentage of community abundance of Proteobacteria was almost similar in control + 1.5% dose of difenoconazole (NG), *T. controversa* + 3% dose of difenoconazole (IB), control + 3% dose of difenoconazole (NH), control + 5% dose of difenoconazole (NI), *T. controversa* + recommended dose of difenoconazole (ID), control + recommended dose of difenoconazole (NJ), control + 1.5 times dose of difenoconazole (NK), and *T. controversa* + no difenoconazole (IF) samples. We also found that for *T. controversa* + 1.5% dose of difenoconazole (IA), *T. controversa* + 3% dose of difenoconazole (IB), *T. controversa* + 5% dose of difenoconazole (IC), and *T. controversa* + recommended dose of difenoconazole (ID), Chloroflexi and Patescibacteria were higher in the infected samples than in the control samples, while Acidobacteria and Actinobacteria were less than in the control samples. However, a reverse scenario was observed in the non-infected samples and samples infected with 1.5 times the dose of fungicide (IE). These findings indicated that with the increase in the concentration of fungicide, the population of Acidobacteria and Actinobacteria also increased, while that of Chloroflexi and Patescibacteria decreased. Following infection with *T. controversa*, the number of Proteobacteria decreased, while that of Acidobacteria and Chloroflexi increased.

Unlike the bacterial communities (Figure 3B), the fungal communities were predominated by four dominant phyla (Ascomycota, Basidiomycota, Mortierellomycota, and Oipidiomycota) with some fungi belonging to the unclassified kingdom. The relative abundance of Ascomycota was high in NL (control + no difenoconazole) and IF (*T. controversa* + no difenoconazole) samples compared to the samples treated with different concentrations of difenoconazole. Basidiomycota was the second most abundant phylum, with the highest relative abundance in *T. controversa*-infected samples, that is, IF (*T. controversa* + no difenoconazole) and IB (*T. controversa* + 3% dose of difenoconazole) samples. The relative abundance of Mortierellomycota and Oipidiomycota was high in IC (*T. controversa* + 5% dose of difenoconazole) and IB (*T. controversa* + 3% dose of difenoconazole) samples. We found that IC (*T. controversa* + 5% dose of difenoconazole) treatment highly decreased the Basidiomycota population, which may contain the pathogen of *T. controversa*.

### Microbial Community Structure in Different Concentrations of Difenoconazole-Coated Seeds in Rhizosphere After *Tilletia controversa* Infection

We observed the resemblances in the microbial communities between samples using Principal Component Analysis (PCA) of weighted UniFrac distances that compare microbial communities based on the phylogenetic relationship. For the infected and control of different treatments, we found IE (*T. controversa* + 1.5 times dose of difenoconazole), IA (*T. controversa* + 1.5% dose of difenoconazole) and IF (*T. controversa* + no difenoconazole) was far away from control, while IB (*T. controversa* + 3% dose of difenoconazole) and IC (*T. controversa* + 5% dose of difenoconazole) had overlap with control which means they were similar (Supplementary Figure 2c). Similarities were examined in fungal communities by using PCA analysis. The control sample (no fungicide) was placed far away from the other samples, which indicated that the inoculation of *T. controversa* significantly changed the composition of the microbial community (Supplementary Figure 2D). We found that the sample infected with 5% fungicide and the control sample overlapped the most, which means this concentration was optimal for the control of the disease.

### Microbial Abundance With Different Concentrations of Difenoconazole Against *Tilletia controversa*

**FIGURE 4:**
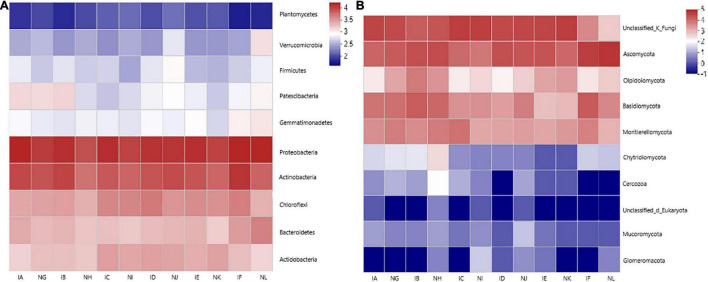
**(A)** Bacterial distribution of the top 10 abundant phyla among the 12 samples. The heatmap plot shows the relative percentage of each bacterial phylum (variable clustering on the *Y*-axis) within every sample (*X*-axis clustering). The percentage value of each phyla is shown by color intensity, and the legend is given on the right side of the figure. **(B)** Fungal distribution of the top eight abundant phyla among the 12 samples. The heatmap plot shows the relative percentage of each fungal phylum (variable clustering on the *Y*-axis) within every sample (*X*-axis clustering). The percentage values of all phyla are shown by color intensity, and the legend is given on the right side of the figure.

Ten bacterial phyla were selected in each sample for clustered heatmap analysis (Figure 4A). Five phyla were found to be abundant in 12 samples, which include, Proteobacteria, Actinobacteria, Chloroflexi, Acidobacteria, and Bacteroidetes, while the remaining five phyla were relatively less abundant in all the samples, which include Planctomycetes, Verrucomicrobia, Firmicutes, Patescibacteria, and Gemmatimonadetes. The members of Proteobacteria, Actinobacteria, Chloroflexi, Bacteroidetes, and Acidobacteria were higher than observed in control, and Patescibacteria population was similar in IA (*T. controversa* + 1.5% dose of difenoconazole) and IB (*T. controversa* + 3% dose of difenoconazole) samples, while significant difference was observed for IC (*T. controversa* + 5% dose of difenoconazole) sample, which indicated that this concentration of fungicide decreased the microbial community of Patescibacteria.

Eight fungal phyla were also selected in each sample for clustered heatmap analysis. The results showed that all 12 samples had a similar and more abundant distribution of Ascomycota, with minor variation among the samples. The abundance of Basidiomycota and Mortierellomycota was similar in all the samples, while the abundance of Olipidiomycota was more in the IB (*T. controversa* + 3% dose of difenoconazole) sample when compared to other samples. On the other hand, the abundance of Glomeromycota, Mucoromycota, Cercaria, and Chytridiomycota was relatively low in almost all the samples. The members of Ascomycota, Oipidiomycota, Basidiomycota, and Mortierellomycota showed significant differences, but the abundance of Basidiomycota in the IC (*T. controversa* + 5% dose of difenoconazole) sample was lower than observed in control, which means that this concentration of fungicide can decrease the population of *T. controversa*.

The study results showed that the abundance of Actinobacteria and Chloroflexi was maximum when compared to the members of other phyla. The abundance of Actinobacteria was the highest in IF (*T. controversa* + no difenoconazole) and IB (*T. controversa* + 3% dose of difenoconazole) samples when compared to non-inoculated samples (Supplementary Figure 3A). In the case of fungi, phylum Ascomycota showed maximum abundance when compared to the other phyla, and maximum abundance was observed in the NL (control + no difenoconazole) sample (Supplementary Figure 3B). Additionally, samples were verified by using a specific primer of *T. controversa* after difenoconazole application.

## Discussion

Our main hypothesis is that the rhizosphere microbial communities will change after infection with *T. controversa* and play a role in the disease incidence and that some of these microbial communities have potential antagonist effects and contribute to the control of the disease. Additionally, the composition of the rhizosphere microbial communities undergoes a drastic change after treatment with difenoconazole fungicide in *T. controversa*-infected and control plants. The morpho-physiological characteristics and performance of a plant are closely linked to various plant-associated soil microorganisms in various conditions (Agler et al., 2016). Many studies have been conducted to determine how microbes help in controlling plant diseases. Several thousand species of nematodes, fungi, protists, and bacteria, including plant root-associated species, can be used as potential antagonist agents against different plant pathogens (Berendsen et al., 2012).

Based on the microbiome analysis, the members of the phyla Bacteroidetes, Actinobacteria, Acidobacteria, Chloroflex, and Proteobacteria were found to be dominant in all the soil samples. These five phyla have been reported to be the dominant phyla in the plant rhizosphere regions of oak, potato, sugar beet, cactus, maize, and *Arabidopsis* (Mendes et al., 2011). The loss of soil microbial diversity due to biotic or abiotic factors contributes to an increase in soil-borne plant pathogens (Van Elsas et al., 2002; Mendes et al., 2015). The high microbial functional diversity and activity are involved in soil-borne disease suppression, plant growth promotion, and plant defense (Jaiswal et al., 2017). Fungi and bacteria are the main components of the plant microbiome, and interactions between these organisms are important in influencing the environmental microbial communication and have vital effects on colonization and viability, thus playing a key role in the pathogenesis of various crop plants (Wargo and Hogan, 2006). The communication and interactions among fungi and bacteria occur via cooperative metabolism, antibiotic production, chemotaxis, protein secretion, molecular signaling or even gene transfer, and various types of biological processes involving different levels of cooperation and antagonism (Frey-Klett et al., 2011). In our results, the abundance levels of Gemmatimonadaceae and Actinobacteria were more in the *T. controversa*-inoculated cultivars, which may play a role in the control of disease in these plants. Actinobacteria play a role in suppressing *Rhizoctonia solani*, a serious plant pathogen (Mendes et al., 2011), and Gemmatimonadaceae members act as antagonist agents against wilt and blight diseases of tomato (Singh et al., 2017). Previous studies have shown that the plants that are infected with pathogens and lack genetic resistance to soil-borne pathogens may enrich specific microorganisms for disease suppression (Kwak et al., 2018; Wei et al., 2019). Our results also showed that the microbial diversity was significantly different in the *T. controversa*-inoculated and control plants, which implies that *T. controversa* may play an important role in changing the soil microbial community of the rhizosphere. Our study results also showed that Acidobacteria was higher in *T. controversa*-inoculated plants when compared to the control plants and thus support the reports of previous studies that higher microbial diversity is linked to higher resistance to plant infestation and pathogen invasions (Mendes et al., 2011; Hu et al., 2016; Zhou et al., 2019). In this study, 16S rRNA gene analysis was performed to determine the community distribution of the rhizosphere soil microbes and their impact on disease incidence in 16 wheat varieties. Further analysis revealed that the norank_c_Subgroup_6, RB41, norank_f_Gemmatimonadaceae, and *Sphingomonas* were the dominant species in the rhizosphere of control and infected plants (Figure 1). Previous studies have demonstrated that pathogen inoculation affects the microbial community, which might be enriched or depleted after infecting the rhizosphere of tomato plants with *F. oxysporum* (Zhou et al., 2020). Nitrosomonadaceae encodes a low-affinity iron permease that may function under iron-replete conditions (Bollmann et al., 2013). Microscillaceae is a new family, and members of this family are proposed to be present in response to *T. controversa* inoculation. Additionally, the microbial population of Acidobacteria was significantly higher in the *T. controversa*-inoculated plants when compared to the control plants (Figure 2A). Similarly, some of the previous studies have shown that different types of plant pathogens directly or indirectly affect the soil microbial community (Fierer, 2017). A considerable number of bacterial strains, particularly *Pseudomonas* spp., have great potential as biocontrol agents against different plant pathogens (Chen et al., 2018). The phenazines that are most commonly produced by *Pseudomonas* spp. include PCN, PCA, pyocyanin, and hydroxyphenazines, which are crucial for the biocontrol efficiency of bacterial biocontrol agents against various fungal diseases (Chin-A-Woeng et al., 2003; Mazurier et al., 2009). Soil microbes respond to pathogens and play a role in regulating the soil environment (Kwak and Weller, 2013). In this study, we found that the disease incidence was related to the abundance of some microbial populations. When the abundance levels of Acidobacteria increased, the rate of disease incidence also increased. Similarly, in the case of Pyrinomonadaceae, Actinobacteria, and Nocardioides, the disease incidence increased as the abundance levels of the microbes increased. However, with regard to Microscillaceae and *Sphingomonas* species, after a certain period of time, the disease incidence increased with the decreasing abundance of the microbial community. Additionally, an inversely proportional relationship was observed between the members of Saccharimonadales and disease incidence (Figure 2).

For seeds coated with difenoconazole fungicide, the abundance of Proteobacteria was high in NL (control + no difenoconazole), IA (*T. controversa* + 1.5% dose of difenoconazole), and IC (*T. controversa* + 5% dose of difenoconazole) samples, and the relative abundance of Actinobacteria was higher in fungicide-treated soils when compared to the control plants (Figure 3A). This finding suggested that plants susceptible to a particular pathogen tend to increase the population of specific microorganisms that act as biocontrol agents (Mendes et al., 2011; Kwak et al., 2018). The loss of rhizosphere microbial diversity contributes to an increase in the incidence of soil-borne diseases, such as damping-off disease caused by *Pythium sylvaticum*, and black scurf and stem canker of potato caused by *Rhizoctonia solani* AG3 (Van Elsas et al., 2002; Jaiswal et al., 2017). Our results demonstrated that the abundance of bacterial and fungal phyla was high in the control and fungicide-coated plants when compared to the *T. controversa*-infected plants (Figure 3). Our results were supported by other studies which reported that fungicides decrease pathogen infections by stimulating the rhizosphere microbial community (Zhou et al., 2020). The abundance levels of Proteobacteria, Actinobacteria, and Ascomycota were significantly high in *T. controversa*-infected plants that were coated with a fungicide when compared to the control plants (Figure 4), and the above-mentioned findings were supported by the previous studies (Newman et al., 2016; Cardona et al., 2018). Overall, we optimized the fungicide concentration to control dwarf bunt based on the signature of the microbial community.

## Data Availability Statement

The datasets presented in this study can be found in online repositories. The names of the repository/repositories and accession number(s) can be found below: https://www.ncbi.nlm.nih.gov/sra/?term=SRP359013.

## Author Contributions

All authors listed have made a substantial, direct, and intellectual contribution to the work, and approved it for publication.

## Conflict of Interest

The authors declare that the research was conducted in the absence of any commercial or financial relationships that could be construed as a potential conflict of interest.

## Publisher’s Note

All claims expressed in this article are solely those of the authors and do not necessarily represent those of their affiliated organizations, or those of the publisher, the editors and the reviewers. Any product that may be evaluated in this article, or claim that may be made by its manufacturer, is not guaranteed or endorsed by the publisher.

## Footnotes

1
http://drive5.com/uparse/

2
http://rdp.cme.msu.edu/

## Supplementary Material

Supplementary Figure 1 Molecular detection of *T. controversa* from the leaf samples using specific primers. M, DL2000 marker (100, 250, 500, 750, 1,000, and 2,000 bp); line 1, positive control; lines 5, 7, and 11, *T. controversa*-infected leaf samples; lines 2–4, 8–10, and 12, negative controls; black arrows indicate the target band of size 372 bp.

Supplementary Figure 2 **(A)** Shannon curves of different rhizosphere soil bacteria in *T. controversa*-infected and non-infected samples at the phylum level. **(B)** Shannon curves of different rhizosphere fungi in *T. controversa*-infected and non-infected samples at the phylum level. **(C)** Principal component analysis (PCA) of weighted UniFrac distances of bacterial communities. **(D)** Principal component analysis (PCA) of weighted UniFrac distances of fungal communities.

Supplementary Figure 3 **(A)** ANOVA test for bacterial community. **(B)** ANOVA for fungal community.

Supplementary Table 1 Sequence analysis of the samples after T. controversa infection.

Supplementary Table 2 The list of core OTUs of bacterial community in T. controversa infected and non-infected samples after fungicide seed dressings.

Supplementary Table 3 The list of core OTUs of fungal community in T. controversa infected and non-infected samples after fungicide seed dressings.
