# Supplementary figures and images for: Characterization of Rhizosphere Microbial Communities for Disease Incidence and Optimized Concentration of Difenoconazole Fungicide for Controlling of Wheat Dwarf Bunt
Source: Front Microbiol. 2022 May 9;13:853176. doi: 10.3389/fmicb.2022.853176 (PMC9125210; doi:10.3389/fmicb.2022.853176)

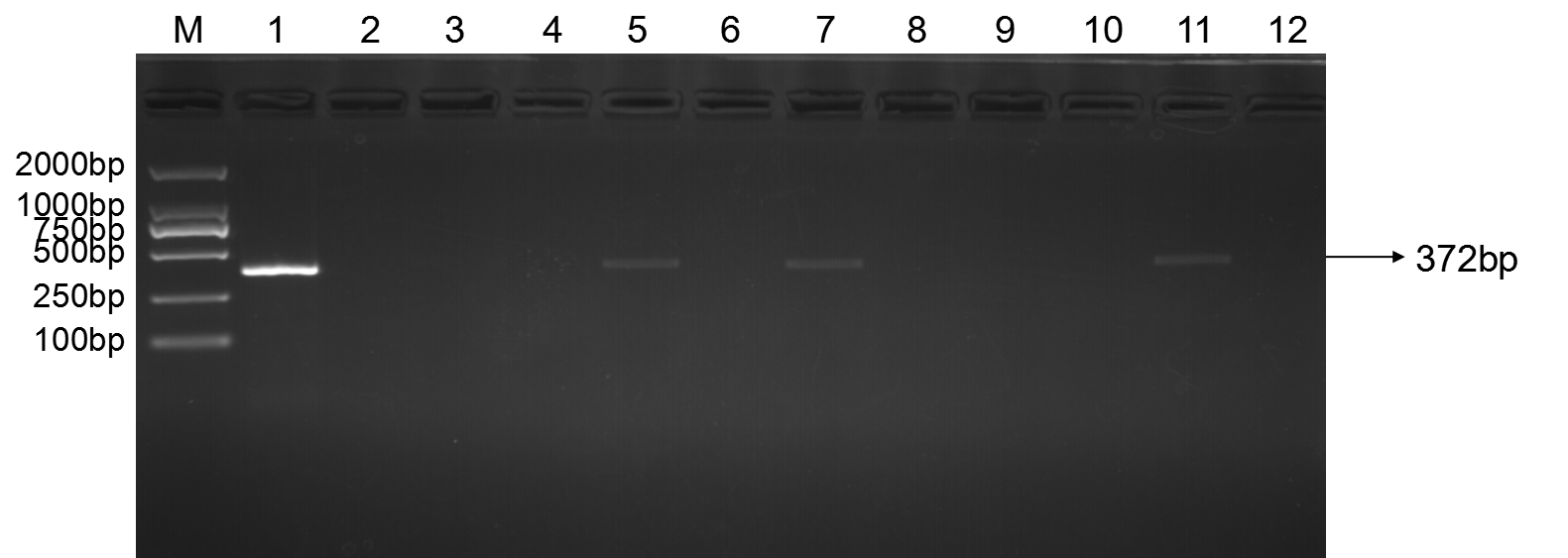

Supplement: Supplementary Figure 1 — Molecular detection of T. controversa from the leaf samples using specific primers. M, DL2000 marker (100, 250, 500, 750, 1,000, and 2,000 bp); line 1, positive control; lines 5, 7, and 11, T. controversa-infected leaf samples; lines 2–4, 8–10, and 12, negative controls; black arrows indicate the target band of size 372 bp. [file Image_1.TIF]

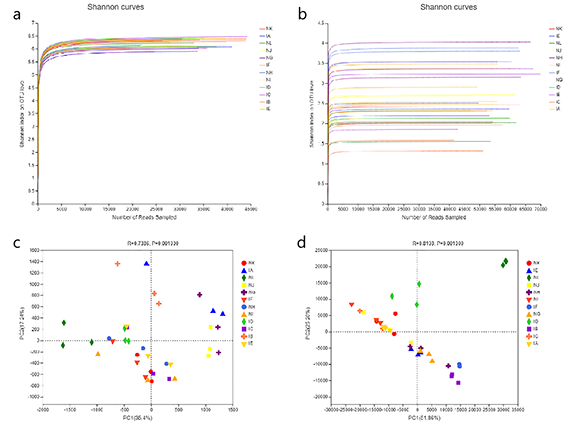

Supplement: Supplementary Figure 2 — (A) Shannon curves of different rhizosphere soil bacteria in T. controversa-infected and non-infected samples at the phylum level. (B) Shannon curves of different rhizosphere fungi in T. controversa-infected and non-infected samples at the phylum level. (C) Principal component analysis (PCA) of weighted UniFrac distances of bacterial communities. (D) Principal component analysis (PCA) of weighted UniFrac distances of fungal communities. [file Image_2.TIF]

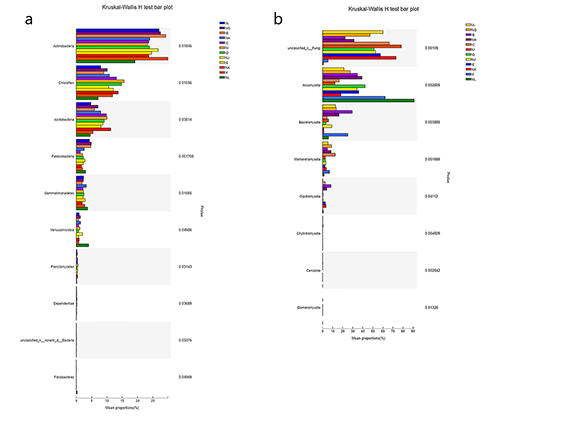

Supplement: Supplementary Figure 3 — (A) ANOVA test for bacterial community. (B) ANOVA for fungal community. [file Image_3.TIF]
